# Editorial: From Stimulus to Behavioral Decision-Making

**DOI:** 10.3389/fnbeh.2019.00274

**Published:** 2020-01-10

**Authors:** Gérard Manière, Gérard Coureaud

**Affiliations:** ^1^Centre des Sciences du Goût et de l'Alimentation, AgroSup Dijon/CNRS UMR6265/INRA UMR1324/Université Bourgogne-Franche-Comté, Dijon, France; ^2^Centre de Recherche en Neurosciences de Lyon, INSERM U1028/CNRS UMR 5292/Université Claude Bernard Lyon 1, Bron, France

**Keywords:** behavior, sensory detection, invertebrates, vertebrates, stimuli

In their natural environments, animals are continuously exposed to sensory complexity, due to the huge number and diversity of stimuli emitted and contained in their surroundings, within and between sensory modalities (somesthesy, olfaction and taste, audition, vision), and due to fluctuating and sometimes unpredictable conditions. Two main kinds of environmental factors have consequences on behavior in both solitary and gregarious species, abiotic factors (e.g., temperature, pressure, salinity, hygrometry, light/darkness, etc.) and biotic factors produced by organisms themselves (movements, displays, calls, colors, pheromones, etc.). These various stimuli regulate the life of animals and their interactions in the ecosystem. The present Research Topic aimed at providing illustrations, reflections, and elements of discussion about some of the stimuli that lead to decision making in vertebrates and invertebrates and about mechanisms that support their emission and/or action.

In response to environmental changes, animals adapt their physiology (homeostasis, etc.) and behavior (food seeking, avoidance of predators, social interactions, etc.) to survive. Responses to environmental stimuli may be either spontaneous, i.e., result from predispositions to perceive and process particular signals, or dependent on experience, i.e., be acquired through simple exposure, or associative/non-associative learning episodes. Learning may contribute to reduce or discard behavioral experiences that have negative consequences, as shown here in the original paper by Yang et al.. They found that the larvae of zebrafish could acquire by operant conditioning a new visual stimulus like pure-black pattern (conditioned stimulus) when it is associated with electric shock (unconditioned stimulus). The contrast of the visual stimulus (grayscale visual pattern) then modulates the learning responses and retention of the visual information in the larvae.

Animal behavior is also regulated by alternating day-night cycles over the nycthemeral period of 24 h, which can lead to the expression of circadian rhythmic behaviors. Some animals indeed have a daytime activity, whereas others have a nocturnal one. A fascinating question is how various sources of information are combined to sense a single time of the day in an animal. Actually, behavioral activities are mediated by internal clocks supported by neurophysiological mechanisms from the peripheral and/or central nervous system. Among these mechanisms, molecular processes may contribute to the internal clock and generation of the locomotor circadian rhythm. The mini-review by Somers et al. shows in particular that in *Drosophila*, locomotor activity depends on two brain oscillators, one that controls the morning locomotor activity and another that governs the evening activity. These clocks are autonomous oscillators, and the phase can be adjusted by light or thermal stimuli.

Animals including humans may currently benefit from five main senses (more or less functional, depending on the species and on the developmental stages) allowing them to detect, discriminate, represent, and respond behaviorally to external stimuli.

Olfactory or gustatory organs in animals allow for detecting chemical cues merging from the ecosystem. These organs are fundamental, e.g., for food searching, identification of conspecifics, recognition of sexual partners, or detecting dangers such as predators or noxious compounds. For instance, some insect species are known to be major pest of crops. Many methods aim to use mating disruption to control insect populations using synthetic sex pheromones. In his review, Cattaneo shows the interest of the identification and functional characterization of the chemosensory receptors of *Cydia pomonella* (Lepidoptera: Tortricidae), a pest of apple, pear, and walnuts. A better knowledge and understanding of the olfactory mechanisms of pests would allow for a better control of their action by using the olfactory system as a target. About odor-guided searching for food, de Fouchier et al. show in invertebrates that caterpillars from a phytophagous insect, the moth-model pest *Spodoptera littoralis* (Noctuidae), respond behaviorally to plant volatiles. These chemical cues are ligands of olfactory receptors identified by the authors. By a complementary modeling approach, the authors pinpoint some receptors whose activation is related to caterpillar attraction. In vertebrates, Pettersson et al. show that attraction to mammalian blood odor is not exclusive to top predators (such as tigers and wolves), but that such an attraction may be also displayed by a small-bodied mesopredator, here the meerkat (*Suricata suricatta*). The study reveals that this attraction is triggered by a single molecule contained in mammalian blood, which seems to be the TED single molecule (trans-4,5-epoxy-(E)-2-decenal).

At the periphery of the olfactory system, olfactory receptor neurons sense odor cues. These sensory inputs are then processed by the brain to promote attraction or avoidance responses. Claßen and Scholtz show that in *Drosophila*, the behavior induced by an olfactory stimulus like ethanol can be shifted depending on specific activation of particular neurons. Indeed, the activation of a cluster of octopaminergic neurons leads to an attraction toward ethanol, while the activation of another group of octopaminergic neurons results in avoidance of the same stimulus. Thus, octopaminergic neurons are directly involved in decision making and contrasted behaviors in *Drosophila*.

Social insects live in colonies and communicate with conspecifics to transmit crucial information carrying adaptive values, e.g., enabling colony provisioning, defense, and reproduction. In hives, bees that have found and located a food source in the surroundings transfer multiple sensory information using the waggle dance to bee dance followers. In the present *Frontiers* Research Topic, Moauro et al. show that bee dance followers have better memory retention and higher gustatory responsiveness to sucrose than non-follower bees. The followers are more sensitive to environmental stimuli compared to non-followers and could decipher more easily the nature and localization of the food source encoded by the bee waggle dance.

Concerning taste and underlying neurophysiological processing, research in animal models may have fundamental impacts allowing for improving knowledge in human nutritional physiology, brain structures, and related behaviors. In their proof-of-concept study, Coquery et al. show that potentially appetitive (sucrose) or aversive (quinine) gustatory stimulations may be combined with functional MRI in minipigs to promote brain responses in part concordant with results already obtained in humans. This may constitute a promising way to run preclinical investigations that compare the integration of gustatory stimuli in healthy individuals or individuals suffering from dysgeusia or eating disorders.

In audition, certain signals carry crucial values in terms of adaptation and survival, as, for instance, in the case of signals that indicate the immediate presence of a predator. Animals must therefore remain attentive in order to select relevant auditory information that helps in deciding for an escape response. In their original study, Chapuis and Chadderton reveal that mice can detect a target sound from another in a sound mixture and that the activity of the auditory cortex is modulated during the different attentional states.

Surviving from threats such as predators depends also in some species on vision, and on acquired or innate defensive responses to visual stimuli. However, the regulation of innate fear responses by the additional perception of pleasantness/unpleasantness still remains debatable. In their original research, Liu et al. test the consequences of gentle handling of the innate response of mice to a visual looming stimulus. They show that handling attenuates the defensive response of mice in correlation with cortical plasticity, as the de-excitation of specific layers of the superior colliculus, and change in the connection between the cortex and the subcortex.

Finally, visual attention is also important in humans, for instance, as a contributor to consumer decision making. While individuals' determinants of this attention remain weakly understood, Audrin et al. propose here an original study assessing how visual attention can be modulated by psychological values in the context of forced-choice experiments proposing luxury/non-luxury ready-to-wear products. They show that participants with high vs. low levels of materialism display with visual attention to distinct information carried by the products (symbolic dimension vs. actual characteristics) that may end in the choice of a same product but for different reasons.

Thus, innate or learned responsiveness to stimuli and consecutive behaviors allow animals to adapt to their ecosystem and to provide for their vital needs such as food searching, sexual interacting with partners, and escaping from predators. To that goal, animals may modify their physiology and behavior by detecting changes in the environment. By being exposed at the same time or sequentially to stimuli from different sensory modalities like olfactory, visual, and auditory events, animals must combine multisensory information to adjust their behavior. The peripheral and central nervous systems play a major role in the integration of this information allowing for making a decision and *in fine* to display a coherent and efficient behavioral response.

## Author Contributions

GM and GC conceived and supervised this Frontiers Research Topic and wrote and edited the editorial.

### Conflict of Interest

The authors declare that the research was conducted in the absence of any commercial or financial relationships that could be construed as a potential conflict of interest.

